# The Removal of Adenoids

**Published:** 1902-06-28

**Authors:** 


### THE REMOVAL OF ADENOIDS.

IN the second of the Ingleby Lectures delivered before the University of Birmingham, Mr. F. Marsh dealt with the various problems presented by adenoid growths in the naso-pharynx. After speaking of the evil effects produced by these growths, he proceeded to consider the methods adopted by various authors for their removal, and described the plan which he himself adopts. An anæsthetic should, he says, always be given, and he lays great stress on the necessity of its being administered by an experienced anæsthetist. On the whole, he prefers chloroform for children, given by dropping it on a single fold of towel, flannel, or lint; but for older children and adults ether or gas may be employed. Kelene or ethyl chloride, however, he thinks likely to supersede gas. It gives on an average about two minutes of anæsthesia and analgesia. The patient should be placed in the recumbent position with the head and shoulders slightly raised on a low pillow. When the desired stage of anæsthesia has been reached, the mouth is opened with a suitable gag on the left side. A large sized, slightly curved Gottstein curette is then introduced behind the palate, the handle being firmly held in the fist—dorsum upwards—and not as a scalpel or pen. The blunt edge is kept well against the septum and passed upwards to the pharyngeal vault until slight resistance is felt by the cutting edge meeting the adenoids; firm pressure is made for a moment, and then a quick *tour de main* is made, taking the handle upwards and the blade downwards. This makes a clean section of the adenoid mass and brings it, if of any size, into or outside of the mouth. The curette is then quickly reintroduced and a supplementary sweep is made by it on each side to clear away any lateral fringe. An examination is now made with the finger to ascertain if the removal has been complete; if so, the tonsils are quickly removed, unless from their size it had been necessary to remove them before the adenoids, and the child is turned well over on his side to allow the free escape of blood through the mouth. The success of the operation depends on the proper engagement of the adenoids by the curette. If the curette is not kept well up to the septum an anterior fringe is very apt to be left, while if the curette is too small crushed lateral pieces are left which may escape the supplementary lateral curettement. If examination with the finger shows that removal is not complete the curette must be used again, or a small pair of Jurac's or St. Clair Thomson's forceps may be substituted.

